# Supplementary material for: Intraspecific sequence variation and complete genomes refine the identification of rapidly evolved regions in humans
Source: Cell Genom. 2026 Sep 23;6(10):101363. doi: 10.1016/j.xgen.2026.101363 (PMC13689511; doi:10.1016/j.xgen.2026.101363)
Supplement: Supplementary file 2 [file mmc6.pdf]

Cell Genomics Transparent Peer Review Record

Intraspecific sequence variation and complete genomes refine the identification of rapidly evolved regions in humans

Author list: Yanting Luo, Riley J. Mangan, Seth Weaver, Sarah A. Zhao, Federica Mosti, Michael C. Thomas, Ravi Karra, Debra L. Silver, Manolis Kellis, Craig B. Lowe

Summary

Initial submission:	Received : October 20th 2025
	Scientific editor: Judith Nicholson
First round of review:	Number of reviewers: 2<br>Revision invited : March 24th 2026<br>Revision received : April 24th 2026
Second round of review:	Number of reviewers: 1<br>Accepted : 24th August 2026
Data freely available:	Yes
Code freely available:	Yes

This transparent peer review record is not systematically proofread, type-set, or edited. Special characters, formatting, and equations may fail to render properly. Standard procedural text within the editor's letters has been deleted for the sake of brevity, but all official correspondence specific to the manuscript has been preserved.

Referees' reports, first round of review

Reviewer 1: Minor revision required.

Reviewer 2: This manuscript presents a sophisticated computational and experimental framework to redefine HAQERs. The central innovation lies in moving away from the reliance on single-reference genome assemblies, which historically confounded lineage-specific evolutionary divergence with intraspecific polymorphism. By leveraging a 20-way alignment of T2T assemblies for humans and great apes, the authors provide a more granular and evolutionarily robust catalog of human-specific regulatory innovation. The study is timely, technically rigorous, and provides significant insights into the genetic architecture of human-specific phenotypic evolution.

The shift toward utilizing T2T assemblies represents the current frontier in comparative genomics. The authors successfully disentangle intraspecific polymorphism from interspecies divergence, a long-standing challenge in the field. The use of probabilistic base representations for ancestral state inference is a major methodological improvement over the deterministic methods often utilized in previous studies.

The computational pipeline, implemented through the Gonomics platform, demonstrates high transparency and analytical depth. The decision to control for ILS and the conservative definition of the human ancestral node show a strong commitment to minimizing false-positive discovery of accelerated regions.

The study avoids the common trap of relying solely on computational predictions. The application of multiplex, scSTARR-seq to validate the regulatory potential of these elements in both developing brain and heart tissues provides strong, evidence-based functional grounding for the genomic findings.

Specific points:

1.The authors candidly acknowledge the lack of African ancestry in the current T2T haplotype set. While this is noted as a limitation in the Discussion, the authors should clarify how this specific sampling bias might influence the human ancestral node reconstruction. Does the inclusion of only European and East Asian haplotypes lead to a systematic underestimation of diversity in regions that might be hyper-variable in African populations? A brief sensitivity analysis or discussion on the potential directional bias in divergence estimates would be beneficial.

2.The manuscript argues for the role of ancient positive selection. Given that HAQERs are enriched in repetitive elements, such as SVAs and VNTRs, known for high mutability, it is difficult to fully decouple elevated mutation rates from true selective sweeps. While the authors performed robust statistical tests, like comparing to RAND, normalization against local mutation rate estimates, the distinction between regions that evolve quickly because they are mutation-prone and regions that evolve quickly because they are under selection, remains nuanced. Strengthening the Discussion on this distinction would improve the clarity of the evolutionary model being proposed.

3.The finding that bivalent state enrichment peaks at specific window sizes (1 kbp for enhancers, 200 bp for promoters) is compelling. However, the manuscript could more explicitly address whether the selection signals remain robust across these different spatial scales. Are the HAQERs identified at the 200 bp scale biologically distinct in terms of their selective pressure compared to those identified at larger windows?

4.In Figure 2A, the log2 fold enrichment values for segmental duplications are quite high. It would be helpful if the text provided more context on the potential confounding effect of gene conversion within these regions, which can often mimic the signatures of rapid evolution in comparative genomics.

Authors' response to the first round of review

Reviewer #2: This manuscript presents a sophisticated computational and experimental framework to redefine HAQERs. The central innovation lies in moving away from the reliance on single-reference genome assemblies, which historically confounded lineage-specific evolutionary divergence with intraspecific polymorphism. By leveraging a 20-way alignment of T2T assemblies for humans and great apes, the authors provide a more granular and evolutionarily robust catalog of human-specific regulatory innovation. The study is timely, technically rigorous, and provides significant insights into the genetic architecture of human-specific phenotypic evolution.
The shift toward utilizing T2T assemblies represents the current frontier in comparative genomics. The authors successfully disentangle intraspecific polymorphism from interspecies divergence, a long-standing challenge in the field. The use of probabilistic base representations for ancestral state inference is a major methodological improvement over the deterministic methods often utilized in previous studies.
The computational pipeline, implemented through the Gonomics platform, demonstrates high transparency and analytical depth. The decision to control for ILS and the conservative definition of the human ancestral node show a strong commitment to minimizing false-positive discovery of accelerated regions.
The study avoids the common trap of relying solely on computational predictions. The application of multiplex, scSTARR-seq to validate the regulatory potential of these elements in both developing brain and heart tissues provides strong, evidence-based functional grounding for the genomic findings.

We thank the reviewer for their thorough and thoughtful evaluation of our manuscript. We are glad that the reviewer recognizes the central methodological advances of this work, including the use of a 20-way T2T alignment to disentangle intraspecific polymorphism from interspecies divergence, the use of probabilistic ancestral state inference, and the conservative controls for incomplete lineage sorting. We also appreciate the reviewer's positive recognition of the importance of complementing computational predictions with functional validation through scSTARR-seq in multiple tissue contexts.
The reviewer's detailed comments have prompted several analyses and revisions that strengthened the manuscript. We address each comment in detail below.

Specific points:

1.The authors candidly acknowledge the lack of African ancestry in the current T2T haplotype set. While this is noted as a limitation in the Discussion, the authors should clarify how this specific sampling bias might influence the human ancestral node reconstruction. Does the inclusion of only European and East Asian haplotypes lead to a systematic underestimation of diversity in regions that might be hyper-variable in African populations? A brief sensitivity analysis or discussion on the potential directional bias in divergence estimates would be beneficial.

We thank the reviewer for their insightful comment. We are enthusiastic for the future availability of T2T genomes capturing greater intraspecific diversity, which will refine investigations of adaptive evolution both before and after the emergence of *Homo sapiens*. As the currently available T2T assemblies are derived from individuals of European and East Asian ancestry, our inference of the human ancestral node does not capture variation present in African populations. This sampling bias warrants additional clarification and discussion.
Of relevance is the potential misidentification of ancestral state probabilities when ancestral alleles would have been observed in T2T genomes with African ancestry, but were absent from the sampled haplotypes. In such cases, a derived allele present in all sampled genomes may be incorrectly inferred to be present in all humans, leading to a modest overestimation of divergence between the reconstructed human-chimpanzee ancestral node and the human ancestral node. This could cause some mutations that occurred after the out-of-Africa migration to be misclassified as fixed changes on the human-lineage and thus contribute to the identification of consensus HAQERs.

Several considerations constrain the magnitude of this effect. First, the evolutionary timescale (~60-90kya since the out-of-Africa migration) in which polymorphisms could fix in out-of-Africa populations is small (approximately 1%) compared to the ~6-7 mya divergence between humans and chimpanzees. Consequently, polymorphisms arising or persisting exclusively within African populations would be small compared to the number of fixation events on the total branch length underlying human-chimpanzee divergence. Second, our ancestral state inference incorporates multiple outgroups (12 great ape haplotypes) and multiple ingroups (8 human T2T haplotypes), reducing the sensitivity to population-specific derived variants and stabilizing ancestral state assignment.

To further clarify the potential impact of unsampled African diversity, we considered three possible allele configurations at a given site:

1. Ancestral allele could have been observed in African populations, derived allele fixed in sampled haplotypes (e.g., ancestral state A; African genomes A; non-African genomes C). In this scenario, the derived allele may be mis-inferred as fixed across humans, leading to a potential overestimation of human-lineage divergence. While this case could potentially inflate divergence estimates and contribute to false-positive inclusion of sites in consensus HAQERs, the following two cases do not. Such sites may be of evolutionary interest nonetheless, reflecting rapid divergence from the ancestral state in some human populations and future studies with a greater sampling of T2T human assemblies will be able to disentangle these scenarios.
2. Derived alleles could have been observed in African populations, with ancestral alleles retained in sampled haplotypes (e.g., ancestral state A; African genomes C; non-African genomes A). In this case, the sampled haplotypes correctly reflect the ancestral state, and no spurious human-lineage substitutions are inferred. Such sites would not have contributed to consensus HAQER identification due to their lack of divergence, so the underestimate of variation within humans would not influence the Consensus HAQER set.
3. Distinct derived alleles in African and non-African populations. (e.g. ancestral state A; African genomes C; non-African genomes G). Here, both populations carry derived alleles, but the site is polymorphic within humans. We quantify divergence between sites using the cosine distance between 4-state probability vectors encoding our confidence in the allele state at an internal phylogenetic node. In this scenario, our current analysis compares the ancestral state vector [1 0 0 0] (fixed A allele) to [0 1 0 0] (fixed C allele). The inclusion of African genomes may update our inference of the human ancestral node to a vector similar to [~0 0.5 0.5 ~0] (uncertainty between the C and G allele), but it will not change that the model is certain a genetic change occurred on the branch leading to all humans.

For clarity, we have added the following sentence in the results section, clarifying our nomenclature and interpretation of the inferred Human Ancestral Node:

Initial Submission: Prompted by this observation, in this study, we define *Consensus HAQERs* as the most divergent regions separating the inferred human-chimpanzee ancestor from an inferred human ancestral node, the most recent common ancestor of all extant human haplotypes in our 20-way alignment (**Figure 1D**).

Revised Submission: Prompted by this observation, in this study, we define *Consensus HAQERs* as the most divergent regions separating the inferred human-chimpanzee ancestor from an inferred human ancestral node of sampled human haplotypes in our 20-way alignment (**Figure 1D**). We note that in the absence of T2T haplotypes from African populations, this inferred node does not comprehensively account for extant human variation, a limitation we consider in *Discussion*.

Furthermore, we have expanded the discussion of this point with the following paragraph:

Initial Submission: "We leveraged available T2T human assemblies, including one European haplotype, two Ashkenazi haplotypes, and five Han Chinese haplotypes. Notably, this set lacks T2T genomes of African ancestry, which are needed to represent a major component of human genetic diversity. The future availability of more diverse T2T genomes will be valuable to further refine the set of regions that consistently show elevated divergence across all humans."

Revised Submission: "We leveraged available T2T human assemblies, including one European haplotype, two Ashkenazi haplotypes, and five Han Chinese haplotypes. Notably, this set lacks T2T genomes of African ancestry, which are needed to represent a major component of human genetic diversity. The exclusion of ancestral states retained in human populations but absent from the sampled haplotypes may lead to a modest upward bias in inferred human-specific divergence, particularly in cases where substitutions arising after the out-of-Africa migration are misinterpreted as fixed differences. However, as the evolutionary timescale separating modern human populations (~60-90 kya) is small relative to the ~6-7 million year divergence between humans and chimpanzees, the contributions of such polymorphisms to total divergence estimates are expected to be limited. The future availability of more diverse T2T genomes will be valuable to further refine the set of regions that consistently show elevated divergence across all humans."

2.The manuscript argues for the role of ancient positive selection. Given that HAQERs are enriched in repetitive elements, such as SVAs and VNTRs, known for high mutability, it is difficult to fully decouple elevated mutation rates from true selective sweeps. While the authors performed robust statistical tests, like comparing to RAND, normalization against local mutation rate estimates, the distinction between regions that evolve quickly because they are mutation-prone and regions that evolve quickly because they are under selection, remains nuanced. Strengthening the Discussion on this distinction would improve the clarity of the evolutionary model being proposed.

We thank the reviewer for considering our position that there need not be mutual exclusivity between mutation and selection. We agree with the reviewer that the distinction between regions that evolve quickly because they are mutation-prone and regions that evolve quickly because they are under selection remains nuanced. For clarity, we have made the following changes in the discussion section.

Initial Submission: "These observations emphasize that regions of elevated mutation rates should be recognized as key substrates of adaptation. While our analyses revealed signatures of ancient positive selection in HAQERs, these analyses were conducted at the level of HAQER sets as a whole. ~~We expect that selection in these regions is heterogeneous, with the divergence in some HAQERs being under selection and other HAQERs evolving neutrally. Developing robust methods to evaluate selection at single loci across deep evolutionary timescales remains an important future challenge~~."

Revised Submission: "We expect that selection across the set of HAQERs is heterogeneous, with the selective pressures acting on each HAQER being different. We also expect some individual HAQERs were not shaped by significant selective forces, and that their large divergence is due to elevated mutation rates and chance. This heterogeneity of selective pressures is also likely to be present at individual positions within an element. Importantly, we do not think these forces of mutation and selection act independently in the genome, but rather that they often cooccur. Regions of the genome producing many variants in a set amount of time may be more likely to produce an advantageous and positively selected variant during a set amount of evolutionary time. A region with such a slow mutation rate that it produces no variants during a period of evolutionary time will not contribute an advantageous variant during that time window or phylogenetic branch. Developing robust methods to evaluate selection and mutation rates at single loci across deep evolutionary timescales remains an important future challenge."

3.The finding that bivalent state enrichment peaks at specific window sizes (1 kbp for enhancers, 200 bp for promoters) is compelling. However, the manuscript could more explicitly address whether the selection signals remain robust across these different spatial scales. Are the HAQERs identified at the 200 bp scale biologically distinct in terms of their selective pressure compared to those identified at larger windows?

We thank the reviewer for raising the question of whether HAQERs identified at different spatial scales represent biologically distinct classes of elements in terms of historic selective pressure, or whether selection signals are robust to window size. To address this question, we extended our analysis of human-ape divergence and polymorphisms (presented in Figure 3 of the original submission), to HAQER sets defined across window sizes spanning 25bp to 1Mbp (**Figure R1**). Additionally, we have included this analysis in the revised manuscript as **Figure S4F-H**.

We quantified the number of human-ape divergent sites, as well as the number of polymorphic sites in the 1000 Genomes Project Phase III dataset, for each set of regions, and normalized to the cumulative length of elements in each set to define divergence density and polymorphism density.

Across window sizes, we observe a monotonic decrease in divergence density with increasing HAQER window length (**Figure R1**, left). This pattern reflects the ascertainment properties of divergence based detection, as smaller windows require highly concentrated clusters of substitutions to achieve genome-wide statistical significance, whereas larger windows can be detected as HAQERs with more moderate elevations in divergence rates.

In contrast, human polymorphism density remains comparatively stable across HAQER sets and is consistently depleted relative to random genomic regions (**Figure R1**, middle). Consistent with this observation, the ratio of polymorphic to divergent sites (one metric associated with selection), is more pronounced at smaller window sizes, suggesting more moderate selective forces on HAQERs ascertained at large window sizes (**Figure R1**, right). All HAQER sets demonstrate a significant deviation in this ratio relative to random elements consistent with historic positive selection, and HAQERs defined at larger window sizes do not appear to represent a fundamentally distinct evolutionary class.

We interpret the progressive attenuation of selection signals at larger scales as consistent with a model of heterogeneous selection in these elements, in which selection acts on short elements interspersed within neutrally evolving sequences within large HAQERs. Large HAQERs may be interpreted as *HAQER hotspots*, or genomic regions where many fast evolving elements are found. This interpretation is supported by **Figure S4D** of the manuscript, which shows an example of a large-scale HAQER composed of multiple smaller-scale HAQERs. Quantifying this further, we found that the 7 HAQERs ascertained at the 1Mbp window size across the genome collectively contain 341 consensus HAQERs identified at the 500bp window size.

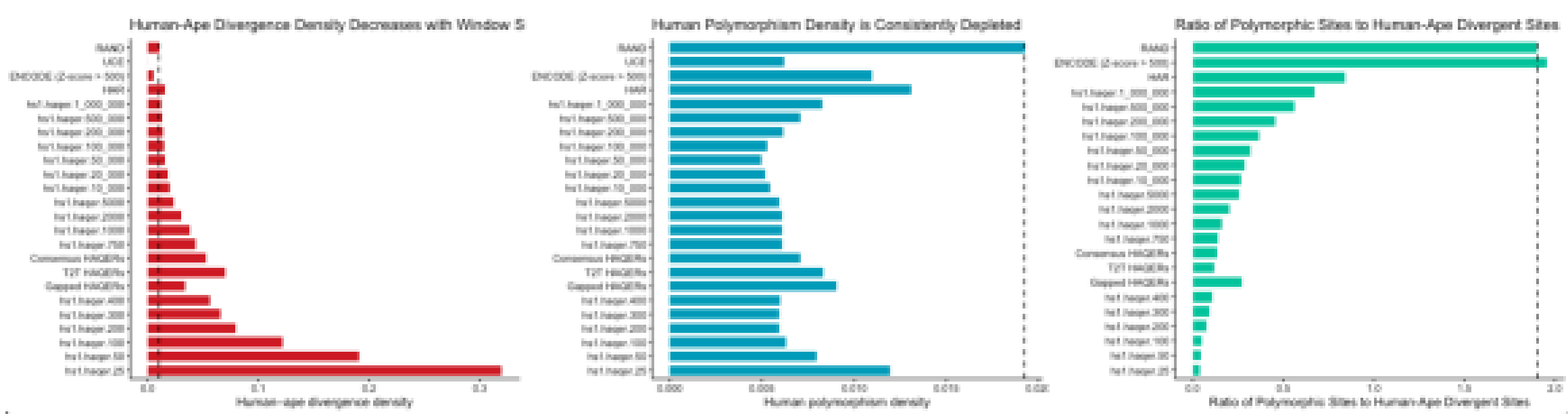

Figure R1: Signatures of selection in HAQERs are robust across spatial scales. (left) Density of human-ape divergent sites across HAQER sets ascertained at window sizes ranging from 25 bases to 1mb. (middle) Density of polymorphic sites among modern humans from the 1000 Genomes Project across region sets show consistent depletion for polymorphic sites across HAQER sets, irrespective of window size. (right)

The ratio of polymorphic sites to human-ape divergent sites, a metric associated with selection, is observed consistently across HAQER sets ascertained at different window sizes.

To reflect these additional analyses, we have made the following additions to the manuscript:
In Results, line 321:

- "Notably, signatures of ancient positive selection, including a reduced ratio of polymorphic sites to human-ape divergent sites, persist in HAQERs ascertained across spatial scales (Figure S4F-H)."

In methods, line 977, a sentence clause has been added to acknowledge the procedure by which divergence and polymorphisms were measured in HAQERs across spatial scales:
For the analysis on hs1, the following region sets were lifted from hg38 to hs1 (see 'Lifting regions across genome assemblies'): gapped HAQERs, HARs, ENCODE, UCEs, hg38 RAND.

- "We also conducted the analysis on hg38 using previously described methods, lifting over the following region sets from hs1 to hg38 (see 'Lifting regions across genome assemblies'): T2T HAQERs, Consensus HAQERs, hs1 RAND, as well as consensus HAQERs ascertained across window sizes ranging from 25bp to 1mb."

In the legend of Figure S4 (line 1214):

- "(F) Density of human-ape divergent sites across HAQER sets ascertained at window sizes ranging from 25 bases to 1mb. (G) Density of polymorphic sites among modern humans from the 1000 Genomes Project across region sets show consistent depletion for polymorphic sites across HAQER sets, irrespective of window size. (H) The ratio of polymorphic sites to human-ape divergent sites, a metric associated with selection, is observed consistently across HAQER sets ascertained at different window sizes."

4.In Figure 2A, the log2 fold enrichment values for segmental duplications are quite high. It would be helpful if the text provided more context on the potential confounding effect of gene conversion within these regions, which can often mimic the signatures of rapid evolution in comparative genomics.

We appreciate the reviewer pointing out the importance of mentioning gene conversion. We also agree with the reviewer that this mutational process can mimic aspects of selection by leading to increased divergence and can be confounding in many analyses. We have added a paragraph to the discussion that: 1) points out to the reader that gene conversion is likely occurring in many HAQERs, 2) that this mutational process can cause elevated divergence without selection, and that 3) there could also be selection acting in these regions and we cite a recent preprint on bioRxiv showing that some HAQERs in regions of biased gene conversion function as gene regulatory elements in primary human cortical progenitor cells using CRISPR interference. The text of the paragraph we added is:
"Functional assays, combined with analyses of mutation and selection, can lead to a better understanding of how various mutational mechanisms contribute to advantageous functional changes. HAQERs tend to occur in segmental duplications, which has likely exposed many of them to the mutational mechanism of gene conversion. Gene conversion can lead to significant divergence without selection, but does not rule out selection also acting in the regions[18,84]. Recent work has noted that GC-biased gene conversion may have contributed to the function of many HAQERs[85]. The authors demonstrated through CRISPRi perturbations that a subset of HAQERs active in human cortical progenitors also show signatures of both biased gene conversion and selection, and propose that the increase in CpG density in the region supported the emergence of gene regulatory function during human evolution[85]."

In terms of additional changes to the manuscript, we corrected the typos we noticed in the manuscript and also marked them red.

Line 400: We corrected a reference to figure panels. (Figure 4D-E (incorrect) now listed as Figure 4B-C).

Additionally, raw and processed sequencing data has now been deposited in GEO, reflected in the following manuscript sentence:

Revised Submission (new text in bold, removed text in strikethrough): **Raw and processed sequencing reads have been deposited at GEO and made publicly available (GEO: GSE311407).** Additional software, as well as raw and analyzed datasets, including browser tracks, sequencing files, multiple alignments, variant sets used in selection analysis, and figure generation pipelines, will be made available at the time of publication on our lab website at https://www.vertgenlab.org. ~~Additional raw and analyzed datasets will be deposited at GEO, to be made publicly available as of the date of publication.~~

We have also updated our citation of Casten et al (citation 32), which previously cited the BioRxiv preprint, to reflect its recent publication in *Science Advances*.

Finally, we updated 2 figures. To Figure 5F, we added a dotted line for negative controls. We also added Figure S4F-H for HAQERs ascertained across spatial scales.

Referees' report, second round of review

Reviewer 2:

The authors have provided detailed responses to all comments and have made substantive revisions to the revised manuscript. The overall quality of the revision is high, and the vast majority of concerns have been adequately addressed.

1.The authors provided a detailed analysis of three allele configuration scenarios, with clear logic. Revised the phrasing from inferred human ancestral node to inferred human ancestral node of sampled human haplotypes, which is more precise. Added quantitative discussion in the Discussion section on the direction and magnitude of bias. Quantify in the Limitations section the approximate proportion of fixed polymorphic sites in out-of-African populations that might be misclassified as human-specific fixed differences. Consider performing a simple sensitivity analysis using African HapMap data, even though it is not T2T, to test whether the core conclusions remain robust under existing data that captures African diversity.

2.Added Figure R1 / Figure S4F-H, covering window sizes from 25 bp to 1 Mb. Found that divergence density decreases monotonically with increasing window size, while polymorphism density remains stable. Concluded that there is no fundamental categorical difference between small-window and large-window

HAQERs. Reinterpreted large HAQERs as HAQER hotspots. Could the Consensus HAQERs (500 bp) enriched within HAQER hotspots (1 Mb windows) be extracted and analyzed separately for their functional characteristics? This would help validate the hotspots model proposed by the authors.

3.Added a new Discussion paragraph acknowledging that gene conversion can elevate divergence without accompanying selection signals. Cited relevant results from Haas et al. 2024. Noted that CRISPRi experiments have validated the regulatory function of some HAQERs in cortical progenitors.

Also cite methodological literature on the impact of gene conversion on sequence evolutionary rates to provide a more comprehensive discussion of the interaction between gene conversion as a neutral/nearly neutral process and selection signals. Provide a rough estimate of the proportion of Consensus HAQERs that are likely to be significantly affected by GC-biased gene conversion.

4.Only 5 HAQERs overlap between the two experiments. Suggest discussing in the Discussion section the recall rate of the screening strategy based on bivalent chromatin and open chromatin. Could this approach have missed a substantial number of functional HAQERs?

5.The brain enhancer assay used mouse embryonic cortex, not human tissue. While the authors have methodological justification, they should more explicitly discuss the limitations of inferring human-specific enhancer activity from a cross-species approach.

6.Figure 1G shows that a considerable proportion of Consensus HAQERs do not overlap with Gapped HAQERs or T2T HAQERs. What are the functional characteristics of these novel Consensus HAQERs? Are they randomly distributed across the genome, or do they possess other structural features?

7.The authors attribute this to the shorter branch length of gorilla, leading to more conservative detection thresholds. However, does this imply that adaptive evolution in the gorilla lineage has been substantially underestimated? Recommend adding discussion of this methodological implication in the Discussion.

8.HAQERs are enriched in repetitive regions, yet the manuscript lacks sufficient discussion of how alignment biases in these regions affect allele frequency estimation. In repetitive regions, short-read mapping biases could lead to systematic underestimation of MAF, which in turn affects the interpretation of

the low polymorphic-to-divergent ratio as a signal of selection.

Recommend adding relevant discussion.

Authors' response to the second round of review

Reviewer's Comments:

Reviewer #2: The authors have provided detailed responses to all comments and have made substantive revisions to the revised manuscript. The overall quality of the revision is high, and the vast majority of concerns have been adequately addressed.

We thank the reviewer for their careful reading of our revised manuscript, as well as our comments describing the revisions. The reviewer has provided a second collection of thoughtful comments which we believe have led to a substantially improved manuscript. In the manuscript, parts of the text that are new or updated appear in red font. We discuss each comment below point by point.

1.The authors provided a detailed analysis of three allele configuration scenarios, with clear logic. Revised the phrasing from inferred human ancestral node to inferred human ancestral node of sampled human haplotypes, which is more precise. Added quantitative discussion in the Discussion section on the direction and magnitude of bias. Quantify in the Limitations section the approximate proportion of fixed polymorphic sites in out-of-African populations that might be misclassified as human-specific fixed differences. Consider performing a simple sensitivity analysis using African HapMap data, even though it is not T2T, to test whether the core conclusions remain robust under existing data that captures African diversity.

We thank the reviewer for suggesting ways to incorporate African diversity data into our HAQER identification pipeline. To quantify the approximate proportion of fixed polymorphic sites in out-of-Africa populations that might have been misclassified as human-specific fixed differences, we adjusted the HAQER identification process by incorporating the hs1-aligned haplotype-phased genotype data from the 501 African individuals gathered by the 1000 Genomes Project. Specifically, we revisited the divergent positions in each Consensus HAQER, where a substitution was inferred between the human-chimpanzee ancestral node and the human ancestral node of sampled haplotypes. Such divergent positions were marked as segregating if the most likely base at the human-chimpanzee common ancestor node appears at >1% frequency in the genotypes of the 501 African individuals. The number of divergent segregating sites per 500 bp in each HAQER was subtracted from the originally identified number of mutations in a 500 bp window to calculate an adjusted divergence count for each HAQER. Of the 1596 Consensus HAQERs, 35 had fewer than 29 mutations in a 500 bp window after the divergence adjustment. For these 35 Consensus HAQERs, incorporating African diversity reclassified enough divergent sites as still-segregating that they no longer met the 29-mutation threshold, indicating they may represent false positives driven by out-of-Africa allele fixation.

Our core conclusions remain robust under existing data that capture African diversity. After adjusting the HAQER identification process by incorporating data from the 501 African individuals in the 1000 Genomes Project, 1561 out of 1596 Consensus HAQERs (97.8%) still had 29 or more inferred human-specific fixed divergences in a 500 bp window. In this 1561-element adjusted-divergence HAQER subset, the enrichments for bivalent enhancers and TSS persist at similar levels as the full Consensus HAQER set, consistent with the large overlap between these sets.

We have added the following text to the Results, Supplemental Figure Legends, and Methods.

Added in Revised Submission (Results): This bivalent enrichment of Consensus HAQERs also persists when taking into account segregating variants in African populations that are not currently represented in T2T human genome assemblies (**Figure S5D, Methods**).

Added in Revised Submission (Supplemental Figure Legend):

Figure S5: Expanded epigenomic analysis of HAQERs, and multi-scale divergence landscapes of the human genome, related to Figure 4

(D) Overlap enrichments between bivalent regulatory states (Bivalent Enhancer; Bivalent/poised TSS) across 127 reference epigenomes[1] and the subset of Consensus HAQERs defined at an adjusted divergence threshold (≥29 mutations after accounting for African population diversity), compared against the full set of Consensus HAQERs.

Added in Revised Submission (Methods):

Adjusted-divergence Consensus HAQER subset

Additionally, we generated an adjusted-divergence Consensus HAQER subset that takes into account existing data that capture African diversity. We identified divergent positions in each Consensus HAQER, where a substitution was inferred between the human-chimpanzee common ancestor node and the human ancestral node. Such divergent positions were marked as segregating if the most likely base at the human-chimpanzee common ancestor node appears at >1% frequency in the genotypes of the 501 African individuals gathered by

the 1000 Genomes Project (see Human genetic variation preprocessing). The number of divergent segregating sites per 500 bp in each HAQER was subtracted from the originally identified number of mutations in a 500 bp window to calculate an adjusted divergence count for each HAQER. Only Consensus HAQERs with at least 29 adjusted mutations in a 500 bp window were retained in the adjusted-divergence Consensus HAQER subset.

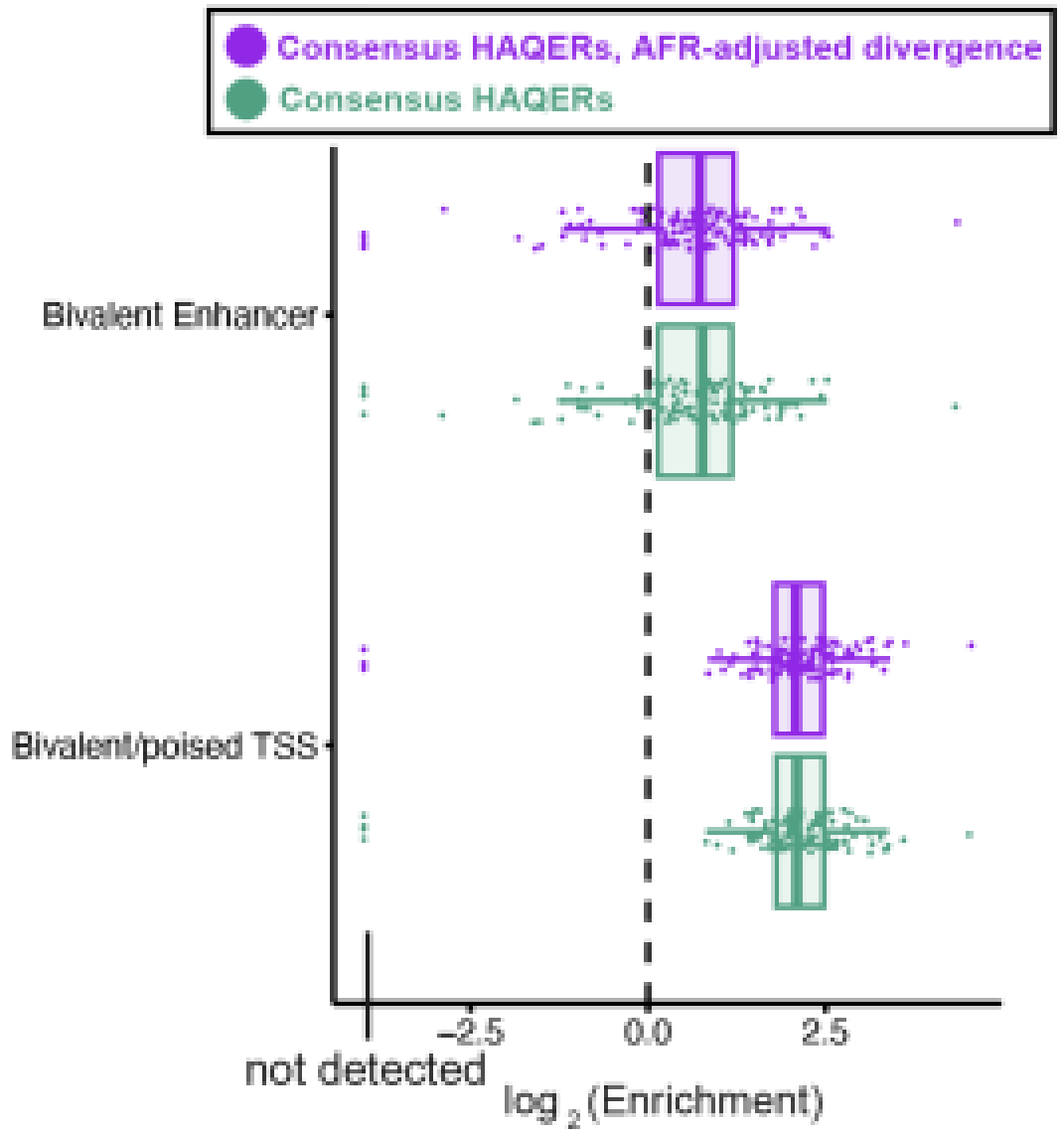

Figure R1: HAQER enrichments for bivalent chromatin states persist when removing elements affected by polymorphisms in African populations.

2.Added Figure R1 / Figure S4F-H, covering window sizes from 25 bp to 1 Mb. Found that divergence density decreases monotonically with increasing window size, while polymorphism density remains stable. Concluded that there is no fundamental categorical difference between small-window and large-window HAQERs. Reinterpreted large HAQERs as HAQER hotspots. Could the Consensus HAQERs (500 bp) enriched within HAQER hotspots (1 Mb windows) be extracted and analyzed separately for their functional characteristics? This would help validate the hotspots model proposed by the authors.

We thank the reviewer for this insightful question. To characterize Consensus HAQERs falling within HAQER hotspots, we intersected the 1,596 Consensus HAQERs (ascertained at the 500bp window size) with HAQERs ascertained at the 1 Mb window size, yielding 107 elements that we term Hotspot HAQERs. When lifted to hg38, this yielded 82 elements, rendering overlap enrichment analysis against bivalent regulatory states underpowered. To have greater statistical power, we created a file of all regions that are bivalent enhancers in any tissue, and a file representing regions that are a bivalent enhancer in any tissue. Overlap enrichments between the hotspot HAQER subset and these files were not significant. This observation points to the hotspots not being hotspots of change in bivalent elements, or at least not in the cell types that have been sampled so far. This is consistent with the hypothesis that very wide windows may capture large-scale fluctuations in mutation rate along the chromosome (or capture a functional change we have not yet identified), while intermediate window sizes (200 bp to 1 kbp) may be the unit sizes of regulatory evolution where sequence divergence is most correlated with gene regulatory divergence.

We then examined their spatial distribution relative to centromeres and telomeres, following the approach described in our response to comment 6. Hotspot HAQERs are strongly enriched within 1 Mb of hs1 centromeric satellite (cenSat) arrays (Fold Enrichment = 4.37, pEnrich = 7.8 × 10-53), a substantially higher enrichment than that observed for Consensus HAQErs overall (fold enrichment = 2.16, pEnrich = 1.92 × 10-93, Figure R2). No Hotspot HAQERs fell within 1 Mb of chromosome ends. Given the small size of the Hotspot HAQER set, this did not constitute a significant depletion (Fold Enrichment = 0, pDeplete = 0.136).

These results indicate that HAQER hotspots are disproportionately located in pericentromeric compartments, consistent with the repeat-dense, mutation-prone nature of this genomic compartment. 1 Mb HAQERs may

reflect local enrichments of the mutational processes that generate individual constituent HAQERs.

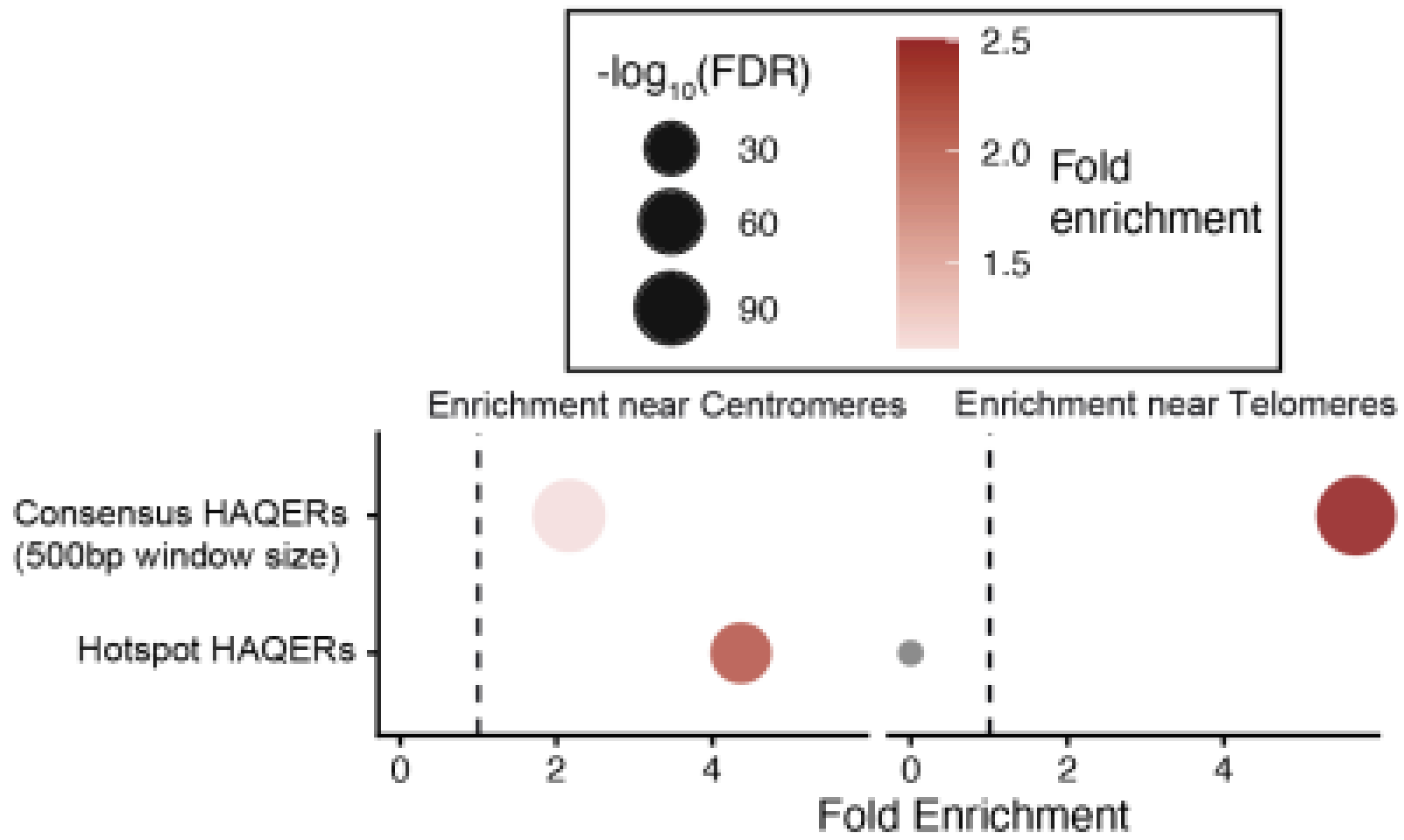

Figure R2: Consensus HAQERs within HAQER hotspots are enriched near centromeric DNA. Overlap enrichments between consensus HAQERs ascertained at the 500bp window size (top) or a subset of consensus HAQERs ascertained at the 500bp window size overlapping HAQERs observed at the 1mb window size (Hotspot HAQERs, bottom). Hotspot HAQERs exhibit greater enrichments near centromeres (left) and were not observed near telomeres (right).

3.Added a new Discussion paragraph acknowledging that gene conversion can elevate divergence without accompanying selection signals. Cited relevant results from Haas et al. 2024. Noted that CRISPRi experiments have validated the regulatory function of some HAQERs in cortical progenitors.

Also cite methodological literature on the impact of gene conversion on sequence evolutionary rates to provide a more comprehensive discussion of the interaction between gene conversion as a neutral/nearly neutral process and selection signals. Provide a rough estimate of the proportion of Consensus HAQERs that are likely to be significantly affected by GC-biased gene conversion.

We agree with the reviewer that it would be helpful to add literature related to gene conversion's effect on the rate of sequence evolution and how gene conversion can mimic signals of selection. We also agree that it would be helpful to include some methods that are attempting to identify regions being influenced by both gene conversion and selection. We have added Glemen et al.[2], which includes evolutionary rates and an excellent discussion of difficulties separating gene conversion and selection. It also makes the important point that, like selection, gene conversion can change where it is acting over evolutionary time. For methods, we have added Capra et al.[3], which identified regions of biased gene conversion and negative selection using a 4-state HMM. We also added Abeykoon et al.[4], which used the ratio of CpGs to GpCs in an attempt to identify regions being acted on by both biased gene conversion and selection.

To estimate the fraction of Consensus HAQERs potentially influenced by GC-biased gene conversion (gBGC), we intersected Consensus HAQERs with the phastBias gBGC tracks, regions predicted to be influenced by gBGC via the phastBias hidden Markov model [3]. We found that 87 of 1,596 Consensus HAQERs (5.5%) overlapped predicted gBGC regions, suggesting that only a minority of Consensus HAQERs are likely to be substantially influenced by gBGC. We note that this should be considered approximate, as currently available phastBias annotations were generated using pre-T2T reference assemblies and subsequently lifted to hs1. Consequently, gBGC tracts within newly assembled centromeric or other previously inaccessible regions are likely underrepresented, and the true proportion may be modestly higher.

We have expanded the main discussion paragraph to now read: HAQERs tend to occur in segmental duplications and near recombination hotspots[5], which has likely exposed many HAQERs to the mutational

mechanism of gene conversion. We note that 87 of 1,596 Consensus HAQERs (5.5%) overlap regions where GC-biased gene conversion is predicted to have occurred[3]. Gene conversion has been associated with highly divergent regions on many vertebrate lineages[6] and it is a significant and ongoing challenge to computationally separate the forces of gene conversion and selection. Gene conversion can lead to rapid sequence divergence and can also quickly change the genomic locations where it acts, mimicking how we might expect strong positive selection to shape a genome[2,7,8]. It is also possible for gene conversion and directional selection to jointly shape regions. More than a decade ago, researchers constructed a statistical framework that could detect gene conversion and purifying selection jointly shaping regions of the genome[3], but detecting both gene conversion and directional selection has continued to be difficult. A recent study noted that both GC-biased gene conversion and directional selection may have contributed to the function of many HAQERs[4]. The authors used CRISPRi perturbations in human cortical progenitors to identify a subset of HAQERs that are functional in that tissue. They then calculated that the subset shows a pattern of forming CpGs compared to GpCs over recent evolutionary time, which might not be expected from purely GC-biased gene conversion acting on those locations[4]. The authors go on to propose that the increase in CpG density in the regions supported the emergence of gene regulatory function during human evolution[4].

Added in Revised Submission (Methods):

GC-biased gene conversion analysis

To estimate the fraction of Consensus HAQERs potentially influenced by GC-biased gene conversion (gBGC), we intersected Consensus HAQERs with the phastBias gBGC tracks, regions predicted to be influenced by gBGC via the phastBias hidden Markov model[3], using gonomics:intervalOverlap[9].

4.Only 5 HAQERs overlap between the two experiments. Suggest discussing in the Discussion section the recall rate of the screening strategy based on bivalent chromatin and open chromatin. Could this approach have missed a substantial number of functional HAQERs?

We fully agree with the reviewer's substantive point: our functional assays were designed to confirm that Consensus HAQERs can act as enhancers in disease-relevant tissues, and were not intended as a comprehensive catalog of functional HAQERs.

We tested 8 HAQERs in the brain and 8 HAQERs in cardiomyocytes. Five HAQER elements exhibited regulatory signatures in brain and heart and were tested in both tissues. Without exhaustively testing all HAQERs for regulatory activity, it is difficult to estimate the proportion of functional HAQERs that could be captured in our assays. However, we can estimate precision. 7 of the 8 (Precision = 0.875) prioritized brain candidates exhibited enhancer activity in developing mouse brains, while 5 of 8 (Precision = 0.625) heart candidates exhibited enhancer activity in cultured human cardiomyocytes. We assayed brain regulatory activity at a single developmental timepoint, and assayed heart regulatory activity in a cultured cell line. If HAQER regulatory activity is restricted to specific cell types or developmental timepoints, these elements may have evaded identification in our approach. As scSTARR-seq is an episomal assay of enhancer activity, elements requiring native genomic context or participating in non-enhancer regulatory functions may have also been missed.

We expect that many HAQERs beyond the 11 distinct elements (8 in brain, 8 in heart, with 5 tested in both) tested here act as regulatory elements for several reasons. First, we only surveyed cell types from two tissues, brain and heart, in the present work based on our group's experimental expertise in measuring enhancer activity in these tissues. Across 833 reference epigenomes, 125 Consensus HAQERs overlap a bivalent enhancer state and 70 overlap a bivalent promoter state in at least one epigenome (**Figure S4A-B**), supporting regulatory function in a much larger fraction of HAQERs. Second, existing reference epigenome catalogs are likely incomplete, with many cell types, developmental time points, and environmental exposures not having been cataloged yet, suggesting that the true number of HAQERs with a gene regulatory function is higher. Third, we conducted our brain tissue experiments in mouse cells, which may lack human-specific cell types or have divergence of the trans-regulatory environment that causes us to not capture the function of some HAQERs. Additionally, we prioritized elements based on regulatory annotations derived from humans, which will not capture human-specific loss of ancestral regulatory elements through sequence divergence. We have added text to the Discussion to make these points explicit.

We note that the five elements assayed in both tissues reflects shared open-chromatin candidates across the two experiments, rather than a measure of assay recall.

Added in Revised Submission: While we have demonstrated the functional relevance of HAQERs in gene

regulation, future work will be required to demonstrate the full breadth of functional change in HAQERs and how HAQER evolution has altered gene expression and organismal phenotypes. Our functional assays were designed to confirm enhancer activity in tissues relevant to human evolution and disease, and do not comprehensively catalog the function of HAQERs. We surveyed cell types from only two tissues (brain and heart) with an episomal enhancer assay, likely missing elements restricted to other cell types, developmental timepoints, requiring native genomic context, or carrying out non-enhancer regulatory functions. Furthermore, we prioritized candidates using human-derived functional annotations, which will not recover elements with human-specific loss of ancestral regulatory functions.

5.The brain enhancer assay used mouse embryonic cortex, not human tissue. While the authors have methodological justification, they should more explicitly discuss the limitations of inferring human-specific enhancer activity from a cross-species approach.

We thank the reviewer for raising this important point. We agree that this assumption is imperfect and that our cross-species approach carries potentially important limitations. Recent studies have demonstrated that trans-regulatory divergence between species is more substantial than previously appreciated, including between humans and non-human primates [10–12]. Consequently, a HAQER sequence assayed in the mouse trans-regulatory environment may not fully recapitulate its activity in the human developing cortex, and elements whose activity depends on human-specific trans factors could be missed. We have added text to the discussion to make these limitations explicit.

At the same time, transgenic and reporter assays in developing mouse embryos have a long history as a standard approach for testing human regulatory element activity[13,14]. This approach rests on the well-supported expectation that, among closely related mammals, trans-regulatory environments are sufficiently conserved that cis-regulatory sequence changes are the primary drivers of gene regulatory divergence[15]. Future work in human and chimpanzee systems will help resolve the relative contributions of *cis*- and *trans*- regulatory divergence to HAQER function.

Added in Revised Submission: Our enhancer assays in the developing brain follow a long-standing approach of testing human regulatory sequences *in vivo* in the developing mouse[13,14], resting on the assumption of a sufficiently conserved *trans*-regulatory environment[15]. As recent studies demonstrate that *trans*-regulatory divergence between humans and other primates is more substantial than previously appreciated[10–12], future studies will help resolve the relative contributions of *cis*- and *trans*-regulatory divergence to functional changes in HAQERs.

6.Figure 1G shows that a considerable proportion of Consensus HAQERs do not overlap with Gapped HAQERs or T2T HAQERs. What are the functional characteristics of these novel Consensus HAQERs? Are they randomly distributed across the genome, or do they possess other structural features?

We thank the reviewer for this insightful suggestion. To investigate the genomic characteristics of the Consensus HAQERs absent from previous catalogs, we examined their spatial distribution relative to centromeres and telomeres (**Figure R3**). Rather than being randomly distributed throughout the genome, newly identified HAQERs are markedly enriched within these genomic compartments.

Consensus HAQERs unique to the present study ("Consensus Unique"; absent from both the original Gapped HAQER set [5] and the T2T HAQER set [16]) exhibited the strongest enrichment near centromeres (3.57-fold enrichment within 1 Mb of hs1 centromeric satellite arrays, $pAdj < 2.5 \times 10^{-88}$). Notably, Gapped HAQERs did not exhibit enrichments near centromeric regions (1.00-fold, $pAdj = 0.49$), consistent with the underpresentation of centromeres in gapped genome assembly-based alignments. Both Consensus HAQERs (2.16-fold, $pAdj < 4.6 \times 10^{-93}$) and T2T HAQERs (1.85-fold, $pAdj < 7.2 \times 10^{-103}$) were significantly enriched in centromeres, with Consensus HAQERs exhibiting more pronounced enrichments than T2T HAQERs. Notably, Consensus Unique and Consensus Not Gapped elements exhibited greater enrichments near centromeres, suggesting that elements newly identified on T2T genomes are disproportionately discovered in regions previously inaccessible to comparative analyses.

To assess HAQER proximity to telomeres, we analyzed overlap enrichments between HAQER sets at regions within 1mb of each chromosome end (**Figure R3C**). As previously described [5], Gapped HAQERs are enriched near chromosome ends (4.24-fold, $pAdj < 1.5 \times 10^{-52}$), a pattern that persists in T2T (4.17-fold, $pAdj < 4.8 \times 10^{-113}$) and Consensus HAQERs (5.69-fold, $pAdj < 1.53 \times 10^{-120}$).

Cell Genomics Transparent Peer Review Record

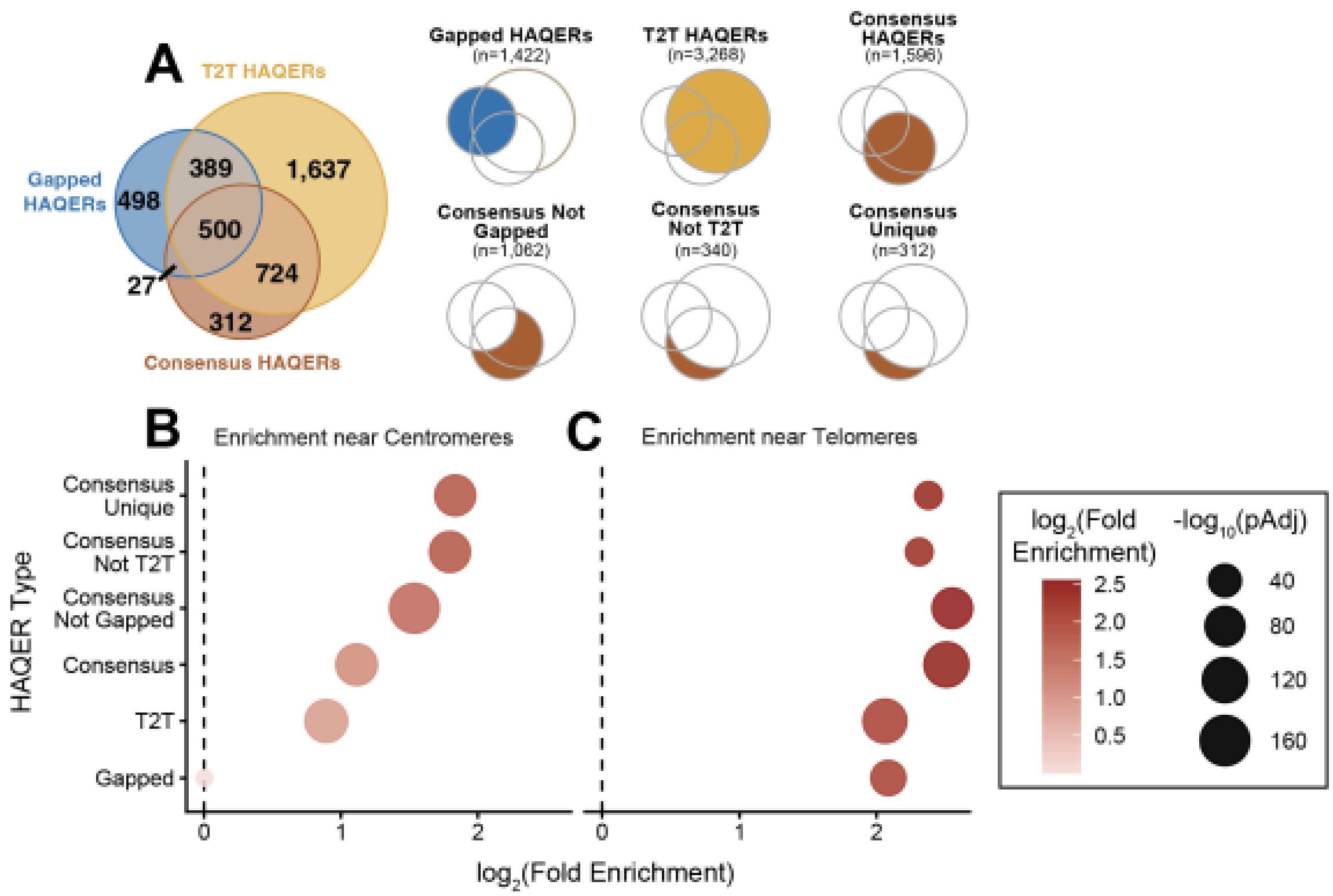

Figure R3: Enrichment of HAQERs near Centromeres and Telomeres. (A) Definitions of HAQER subsets used in this analysis, including Gapped HAQERs, T2T HAQERs, and Consensus HAQERs, as well as Consensus Not T2T, Consensus Not Gapped, and Consensus Unique elements. (B-C) Overlap enrichments between HAQERs and regions within 1mb of (B) centromeric satellite arrays or (C) hs1 chromosome ends, interpreted as telomeres.

To assess whether newly discovered Consensus HAQERs differ functionally from those recovered in previous studies, we examined their enrichment across bivalent chromatin states. The Consensus not T2T (n=340) and Consensus Unique (n=312) subsets were too small for well-powered enrichment analysis. For the larger Consensus Not Gapped subset (n=1,062), we did not observe the enrichment for bivalent enhancer and promoter states seen in the full Consensus HAQER set (**Figure R4**).

We interpret this result cautiously. The chromatin state annotations used here were derived from hg38-based reference epigenomes, which lack much of the centromeric, pericentromeric, and subtelomeric sequence into which the Consensus Not Gapped elements are disproportionately mapped (**Figure R3**). As a consequence, many of these elements fall in genomic regions where hg38-based regulatory annotations are sparse or absent. Therefore, we cannot confidently distinguish lack of bivalent regulatory function from an absence of annotation coverage in these newly accessible regions.

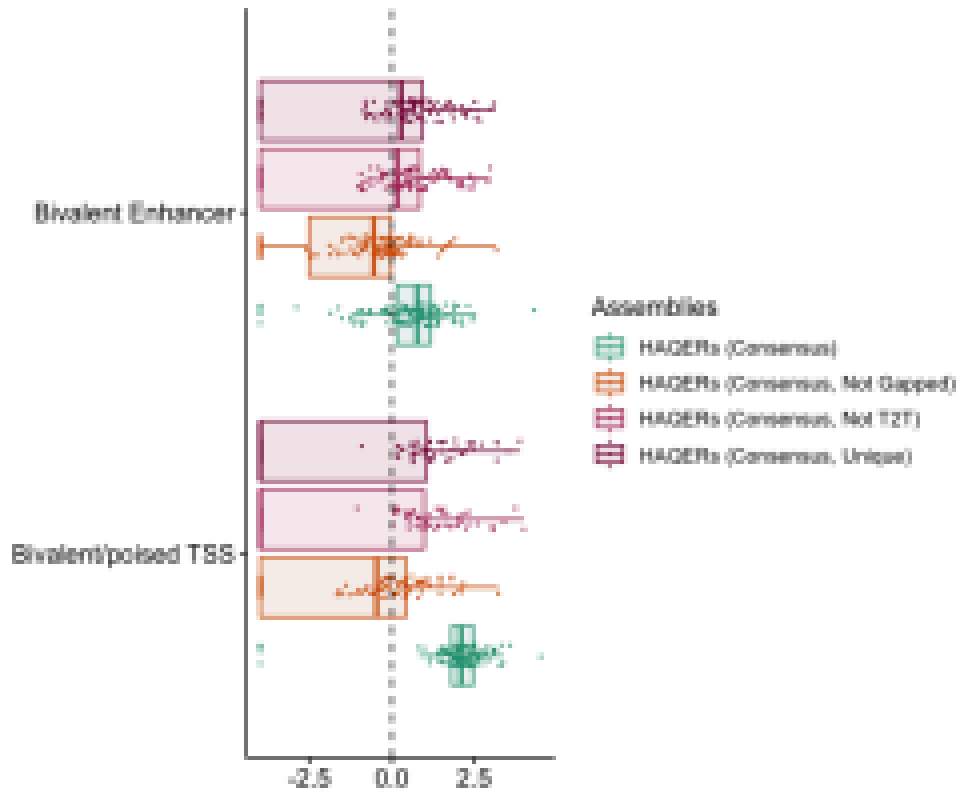

Figure R4: Bivalent chromatin enrichments for newly discovered Consensus HAQER subsets. X axis displays log-scaled fold enrichment for overlaps between each HAQER subset and bivalent chromatin states across reference epigenomes.

Reflecting these analyses, we have included Figure R3 as a new Figure S1 in the manuscript, renumbering all other supplemental figures accordingly. We have added the following text to the results, Supplemental Figure Legends, and Methods.

Added in Revised Submission (Results): We next asked whether Consensus HAQERs absent from previous studies exhibit distinct genomic characteristics, finding these elements to be strongly enriched near centromeres and telomeres (Figure S1). Consensus Unique HAQERs, absent from both the original Gapped and T2T HAQER sets, exhibited a 3.57-fold enrichment within 1 Mb of centromeric satellite arrays, whereas Gapped HAQERs showed no detectable centromeric enrichment, consistent with the underrepresentation of these regions in gapped assembly-based alignments (Figure S1B). Consensus HAQERs were also more strongly enriched near chromosome ends than either previous HAQER set (Figure S1C). These observations indicate that many newly identified rapidly evolved elements reside within genomic compartments that were incompletely represented in earlier reference assemblies.

Added in Revised Submission (Supplemental Figure Legend):

Figure S1: Newly identified Consensus HAQERs are enriched near centromeres and telomeres, related to Figure 1
(A) Relationships among the HAQER sets analyzed, including Gapped HAQERs, T2T HAQERs, Consensus HAQERs, and the Consensus Not T2T, Consensus Not Gapped, and Consensus Unique subsets.

(B) Fold enrichment of each HAQER set within 1 Mb of hs1 centromeric satellite arrays. Consensus Unique HAQERs exhibit the strongest enrichment near centromeres, whereas Gapped HAQERs show no significant enrichment.

(C) Fold enrichment of each HAQER set within 1 Mb of hs1 chromosome ends, a proxy for telomeric and subtelomeric regions. Consensus HAQERs exhibit stronger enrichment near chromosome ends than previous HAQER sets.

Added in Revised Submission (Methods):

Spatial enrichment of HAQERs near centromeres and telomeres
We evaluated whether HAQERs were enriched near centromeres and telomeres using gonomics:overlapEnrichments(Au et al. 2023). We defined human centromeric regions as the hs1 centromeric satellite annotations (cenSat track, UCSC Genome Browser), merged across adjacent intervals and expanded by 1 Mb on each side. We defined telomeric regions as the terminal 1 Mb of each hs1 chromosome arm.

7.The authors attribute this to the shorter branch length of gorilla, leading to more conservative detection thresholds. However, does this imply that adaptive evolution in the gorilla lineage has been substantially underestimated? Recommend adding discussion of this methodological implication in the Discussion.

Our hypothesis is that the *longer* branch leading to gorilla is associated with the smaller number of total

gorilla-AQERs and likely a more conservative detection threshold. While we note the smaller number of gorilla-AQERs, we do not think our study allows for a tightly controlled comparison of the number of AQERs found on different lineages of varying branch lengths.

We have added the following paragraph to the discussion: We detected relatively few gorilla-AQERs, likely impacted by the long phylogenetic branch from the human-gorilla ancestor to extant gorillas. We do not think this result necessarily means that the gorilla lineage has experienced less adaptation or that it has a difference in the molecular mechanisms underlying adaptation. Our framework for identifying functionally significant mutations across lineages may be best tuned for closely related species. For example, a region that experienced rapid evolution for a period of time followed by constraint will no longer appear fast evolving as the branch length, and period of constraint, becomes longer. A number of regions currently detected as HAQERs may no longer be outliers in another 8 million years, using our current method.

8.HAQERs are enriched in repetitive regions, yet the manuscript lacks sufficient discussion of how alignment biases in these regions affect allele frequency estimation. In repetitive regions, short-read mapping biases could lead to systematic underestimation of MAF, which in turn affects the interpretation of the low polymorphic-to-divergent ratio as a signal of selection.

Recommend adding relevant discussion.

We agree with the reviewer that it is difficult to map short sequencing reads to repetitive regions of the genome, and that this difficulty in mapping reads can lead to underestimating the amount of polymorphism in the genome. This would then influence the polymorphism-to-divergence ratio.

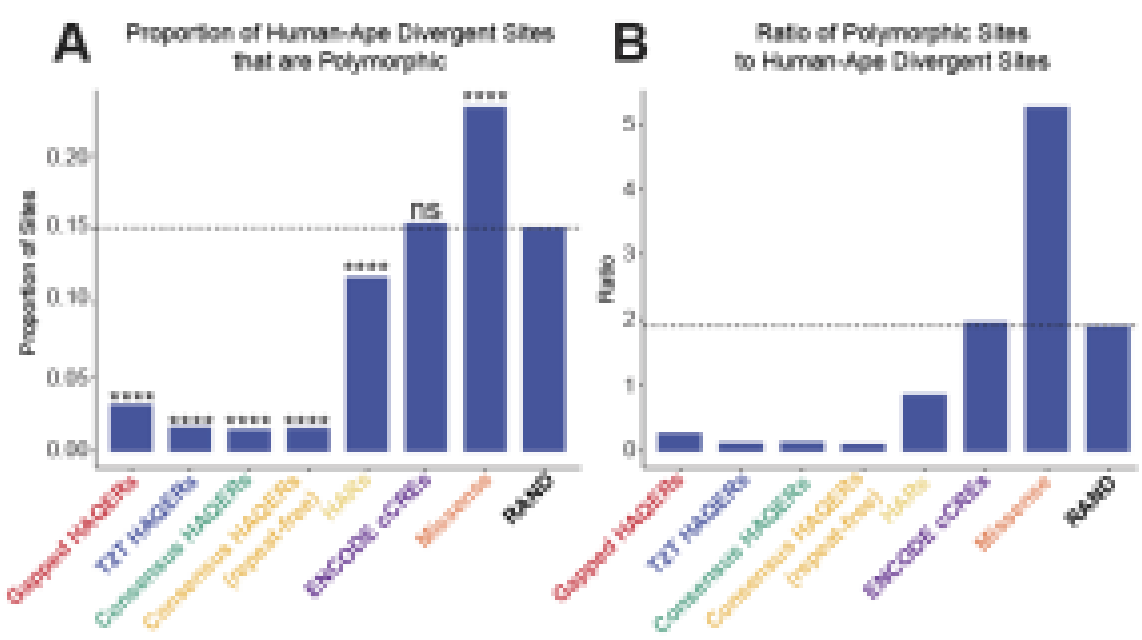

Figure R5: Panels A and B of Figure 3, including polymorphism-to-divergence ratios for repeat-free consensus HAQERs.

To address this, in panels A and B of Figure 3 (reproduced here as **Figure R5**), we not only report this ratio for all Consensus HAQERs, but also the subset of Consensus HAQERs that are free of repetitive regions, which should either reduce or eliminate this bias. We observe a similar polymorphism-to-divergence ratio in the repeat-free subset of Consensus HAQERs, leading us to believe that this concern is not driving the signal in the analysis. However, we agree with the reviewer that this potential bias should be added to the discussion.

Paragraph added to the discussion: The challenges of alignment to repetitive regions is also important to note in the context of detecting human polymorphisms. Short sequencing reads from a human individual are aligned to the reference genome, and the differences are recorded as polymorphisms. Therefore, genomic locations, such as repetitive regions, may appear to be less polymorphic. We did not notice a large difference in polymorphism-related metrics between the entire set of Consensus HAQERs and those that are free of repeats (Figure 3), but we are looking forward to more polymorphism data based on long-read sequencing technologies that will more definitively capture human polymorphisms in technically challenging areas of the genome.

Beyond these changes that were based on reviewer comments, we made the following edits to the manuscript:

- In the Figure 4 legend, there was a mismatch between the panel labels, which has now been corrected and highlighted.
- We made a small cosmetic change to Figure 3, resizing panels to align them with each other.

References

1. Roadmap Epigenomics Consortium *et al.* Integrative analysis of 111 reference human epigenomes. *Nature* **518**, 317–330 (2015).
2. Glémin, S. *et al.* Quantification of GC-biased gene conversion in the human genome. *Genome Res.* **25**, 1215–1228 (2015).
3. Capra, J. A., Hubisz, M. J., Kostka, D., Pollard, K. S. & Siepel, A. A model-based analysis of GC-biased gene conversion in the human and chimpanzee genomes. *PLoS Genet.* **9**, e1003684 (2013).
4. Abeykoon, Y. *et al.* Interrogating the Regulatory Function of HAQERs during Human Cortical Development. *Evolutionary Biology* (2025).
5. Mangan, R. J. *et al.* Adaptive sequence divergence forged new neurodevelopmental enhancers in humans. *Cell* **185**, 4587–4603.e23 (2022).
6. Capra, J. A. & Pollard, K. S. Substitution patterns are GC-biased in divergent sequences across the metazoans. *Genome Biol. Evol.* **3**, 516–527 (2011).
7. Pollard, K. S. *et al.* Forces shaping the fastest evolving regions in the human genome. *PLoS Genet.* **2**, e168 (2006).
8. Kostka, D., Hubisz, M. J., Siepel, A. & Pollard, K. S. The role of GC-biased gene conversion in shaping the fastest evolving regions of the human genome. *Mol. Biol. Evol.* **29**, 1047–1057 (2012).
9. Au, E. H. *et al.* Gonomics: uniting high performance and readability for genomics with Go. *Bioinformatics* **39**, (2023).
10. Gokhman, D. *et al.* Human-chimpanzee fused cells reveal cis-regulatory divergence underlying skeletal evolution. *Nat. Genet.* **53**, 467–476 (2021).
11. Hansen, T. J., Fong, S. L., Day, J. K., Capra, J. A. & Hodges, E. Human gene regulatory evolution is driven by the divergence of regulatory element function in both cis and trans. *Cell Genom.* **4**, 100536 (2024).
12. Song, J. H. T. *et al.* Human-chimpanzee tetraploid system defines mechanisms of species-specific neural gene regulation. *bioRxivorg* (2025) doi:10.1101/2025.03.31.646367.
13. Pennacchio, L. A. *et al.* In vivo enhancer analysis of human conserved non-coding sequences. *Nature* **444**, 499–502 (2006).
14. Visel, A., Minovitsky, S., Dubchak, I. & Pennacchio, L. A. VISTA Enhancer Browser--a database of tissue-specific human enhancers. *Nucleic Acids Res.* **35**, D88–92 (2007).

15. Stergachis, A. B. *et al.* Conservation of trans-acting circuitry during mammalian regulatory evolution. *Nature* **515**, 365–370 (2014).

16. Yoo, D. *et al.* Complete sequencing of ape genomes. *Nature* 1–18 (2025).
